# Validity of the Quarq Cycling Power Meter

**DOI:** 10.3390/s25092717

**Published:** 2025-04-25

**Authors:** Jon Oteo-Gorostidi, Jesús Camara, Diego Ojanguren-Rodríguez, Jon Iriberri, Iván Vadillo-Ventura, Almudena Montalvo-Pérez

**Affiliations:** 1 Faculty of Medicine, Health and Sports, Department of Real Madrid Graduate School, Universidad Europea de Madrid, 28670 Madrid, Spain; jon.oteo@opendeusto.es (J.O.-G.); diegoojanguren@outlook.com (D.O.-R.); 2 Society, Sports and Physical Exercise Research Group (GIKAFIT), Department of Physical Education and Sport, University of the Basque Country (UPV/EHU), 48940 Vitoria-Gasteiz, Spain; 3 AKTIBOki—Research Group in Physical Activity, Physical Exercise and Sport, Department of Physical Education and Sports, Faculty of Education and Sport, University of the Basque Country (UPV/EHU), 48940 Vitoria-Gasteiz, Spain; 4 Visma Lease a Bike Professional Cycling Team, 5215 MV Den Bosch, The Netherlands; j-iriberri@euskadi.eus; 5 Faculty of Medicine, Health and Sports, Department of Sport Science, Universidad Europea de Madrid, 28670 Madrid, Spain; ivan.vadillo@universidadeuropea.es

**Keywords:** power output, laboratory testing, pedaling position, cadence

## Abstract

Technological advancements have led to the development of various devices designed to monitor training loads and athletic performance. Power meters, particularly in cycling, allow for the precise quantification of power output, which is crucial for managing training loads and evaluating performance improvements. This study evaluates the validity of the Quarq D-Zero power meter for measuring cycling power output by comparing it with two previously validated devices—the Favero Assioma Duo (FAD) and the Hammer Saris H3 (H3)—noting that, although it shares the same measurement location as the SRM (the gold standard), it has not been directly validated against it. Thirty-one trained male cyclists participated in this study, undergoing tests across various power outputs (100–500 W) and three 10-s sprint efforts. The protocol incorporated different cadences (70, 85, and 100 revolutions per minute), randomized in order, and two cycling positions (seated and standing). Significant differences (*p* < 0.05) in power readings were observed among the three power meters, except during sprint efforts. However, pairwise comparisons revealed no significant differences (*p* > 0.05) between the FAD and Quarq power meters, except for the 500 W block. Strong to very strong correlations were observed between the FAD and Quarq power meters (*r* > 0.883, ICC > 0.879). The coefficient of variation (CV) between the FAD and Quarq devices ranged from 0.62% to 4.89%, and from 0.39% to 6.59% between the H3 and Quarq power meters. In conclusion, the Quarq power meter, integrated into the spider of the bicycle’s bottom bracket, provides valid power output measurements in cycling.

## 1. Introduction

In Sports Science, there has been a continuous increase in the implementation of technological resources aimed at controlling, monitoring, and precisely quantifying athlete performance and the associated training loads and competitive demands. These tools are fundamental for optimizing sports performance and systematically planning training loads. The range of available technologies is extensive, and their applicability varies according to the specific characteristics inherent to each sports discipline.

In endurance-dominant sports, performance is primarily influenced by the athlete’s ability to sustain maximum power output over the competition-specific distance and by the energy cost associated with maintaining a given race speed [1]. The proliferation of advanced technologies in cycling, characterized by the widespread adoption of power meters, has transformed performance analysis, which serves as a precise indicator of intensity and performance. This significance is attributed to the power’s immediate responsiveness to changes in exercise intensity and its ability to be quantified even at supra-maximal intensities [2]. These devices allow for precise quantification of cyclists’ power output while also providing supplementary data such as cadence, torque, and other critical performance parameters [3]. Over the years, the variety of power meters has expanded significantly, resulting in a wide range of formats designed for power measurement. The market currently provides an increasing number of power meters that incorporate various technologies from multiple manufacturers [4]. This increase has led to a decrease in costs, thereby enhancing accessibility for a larger population of cyclists [5].

The primary function of these devices is to measure an athlete’s power output throughout the pedaling cycle, providing an objective quantification of exercise intensity. Notably, portable power meters can be integrated into several mechanical components of bicycles, including pedals, cranks, chainrings, rear hubs, or the spider of the bottom bracket axle [5]. However, all sensors and technological tools introduced to the market face a critical validation challenge. These devices must demonstrate reliability; therefore, they undergo a validation process involving comparison with a “gold standard” to assess measurement accuracy and repeatability [6]. Many power meters currently in use have undergone comparative validation against the SRM, widely recognized as the gold standard in this field [4].

The Quarq D-Zero is a power meter positioned between the bottom bracket axle and the crankset of the bicycle [7]. Since it shares the same location as the SRM, its validity has not been directly tested against this device. To our knowledge, no validation study of the Quarq D-Zero has been published to date. However, previous research has evaluated the performance of another model from the same manufacturer, the Quarq Quatro [8]. Therefore, this study aims to assess whether the Quarq D-Zero power meter is a valid and reliable tool for measuring power output in cycling.

## 2. Materials and Methods

### 2.1. Experimental Approach to the Problem

A cross-sectional descriptive study was conducted, with recruitment and data collection taking place between February and March 2024. All tests were performed under standardized temperature and humidity conditions (a temperature of 20.3 ± 0.9 °C and a humidity of 28.2 ± 1.3%). Before participation, all subjects received both verbal and written information about the study procedures and provided their written informed consent. Participation in the study was entirely voluntary. The research adhered to the principles of the Declaration of Helsinki and received ethical approval from the Research Commission of the European University of Madrid (CI 2024-524).

### 2.2. Participants

A total of 31 well-trained male participants (age: 33 ± 12.19 years; weight: 70 ± 7.42 kg; height: 1.79 ± 0.06 m) were selected for the study, all of whom engaged in regular and structured training, averaging 11.5 ± 3.9 h per week. Inclusion criteria required participants to be in good physical health, with no known injuries or medical conditions that could interfere with high-intensity cycling performance. Additionally, participants had to be capable of sustaining a power output of 500 W while seated for a continuous duration of one minute.

### 2.3. Procedures

For this study, a bicycle was utilized that could be adjusted to accommodate the measurements of each participating athlete. Both saddle height and frame length were modified to optimize comfort. Three different power meters were affixed to the bicycle for data collection. The first, a Quarq Force D-Zero power meter (Quarq) (SRAM, Chicago, IL, USA), was positioned between the bottom bracket axle and the 170 mm crankset. The second and third devices, Favero Assioma Duo pedals (FAD) (Favero Electronics, Srl., Arcade, TV, Italy) [9,10], were mounted on both pedals, while the Hammer Saris H3 (H3) (CycleOps, Madison, WI, USA) [2] functioned as a cycle-ergometer. Prior to each test, all three devices were calibrated following the manufacturers’ instructions. First, with the crank arm in a vertical position, the Favero and Quarq devices were calibrated. Then, the Saris was calibrated by pedaling until reaching a specific speed and then stopping pedaling, allowing the roller to decelerate on its own. Data from each power meter were collected simultaneously at a frequency of 1 Hz using three Garmin 530 cycle computers (Garmin International Inc., Olathe, KS, USA), which recorded power output in watts (W). Each device (Quarq, FAD, H3) recorded data independently via ANT+ and was later synchronized post hoc using timestamp alignment on a per-second basis, enabling consistent comparison across power meters. To avoid any performance inconsistencies related to battery status, all devices were fully recharged before each testing session. Participants used their cycling shoes, equipped with Look brand cleats.

**Figure 1:**
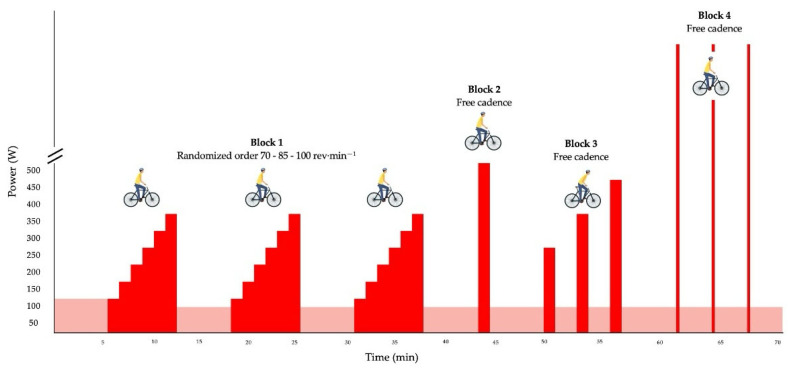
Diagram of the power meter validation protocol. Note: min, minutes; rev·min⁻^1^ revolutions per minute; W, watts.

To validate the Quarq power meter, a protocol like that used in studies such as Montalvo-Pérez et al. (2021) [9] was implemented. This protocol incorporated varying intensities, pedaling cadences, and cycling positions. It began with a 5-min warm-up at 100 W, allowing athletes to select their preferred cadence. Following the warm-up, three identical work blocks were conducted. Each block consisted of six incremental power stages, starting at a submaximal workload of 100 W and increasing to a maximum of 350 W in 50 W increments. The designated cadences (70, 85, and 100 revolutions per minute) were randomized for each cyclist. The first three work intervals (block 1) were performed in a seated position, each lasting 75-s, with 5-min recovery periods at a self-selected cadence and an intensity of 75 W. This was followed by a high-intensity bout (block 2) at 500 W for 75-s, marking the peak intensity of the test. Afterward, three standing intervals of equal duration (block 3) were executed, with power outputs of 250 W for the first, 350 W for the second, and 450 W for the third, interspersed with 2-min recovery periods at 75 W. Following a 5-min rest, the protocol concluded with an all-out sprint phase (block 4), consisting of three maximal 10-s sprints in a seated position, each separated by 2-min recovery periods at 75 W (Figure 1).

Mean power data were collected from all blocks. Of the 75-s measured during the initial three blocks, only 60-s of power and cadence data were analyzed. The initial 10-s and the final 5-s were excluded to ensure the cyclists had sufficient time to stabilize and maintain each designated workload [11]. In the fourth block, which consisted of three 10-s sprints, both the mean power over the 10-s duration and the 1-s power recorded within the first 5-s of the sprint were analyzed [12,13]. To preserve raw signal fidelity, no on-device smoothing or filtering was applied. During post-processing, a 3-s rolling average was implemented to minimize transient noise.

### 2.4. Data Analysis

Data are presented as mean ± SD. The relationship and level of agreement between potentiometers were analyzed with Pearson correlation coefficients (*r*), intraclass correlation coefficients (ICC) (2,1), and standard errors of measurement. The typical error of the Mean (TEM) was calculated as the standard deviation of measurement differences divided by the square root of 2 [14]. Additionally, the coefficient of variation (CV) was also determined. *R* values of 0.1, 0.3, 0.5, 0.7, and 0.9 were considered small, moderate, strong, very strong, and extremely strong, respectively [15]. ICC values less than 0.5, between 0.5 and 0.75, between 0.75 and 0.9, and greater than 0.90 were considered poor, moderate, good, and excellent reliability, respectively [16]. In addition, a two-way repeated-measures ANOVA (power meter [Quarq vs. FAD vs. H3] by power) was conducted to assess differences between the power meters across each cyclist’s pedaling position (sitting/standing) and cadence. To minimize type I error, post hoc Bonferroni tests were only applied when a significant power interaction was found. The magnitude of differences (ES) was analyzed using ηp^2^, with reference values being small (0.01), medium (0.06), and large (0.14). Agreement between power meters was also determined using the Bland–Altman method. The mean difference (bias) and the 95% limits of agreement (bias × 1.96 of the difference) were calculated. Results were graphically examined through Bland–Altman plots, where the differences were plotted against their mean values. The Breusch–Pagan test was performed to assess for heteroscedasticity. Statistical analyses were performed using custom-written software (Python v3.12.7; Python Software Foundation) with an α of 0.05.

## 3. Results

Significant differences (*p* < 0.05) were found between the power measurements of the three power meters, except during all-out sprints. Specifically, in the pairwise comparison between the Quarq and the H3, significant differences in power output were observed across all intervals except for the all-out sprints. On the other hand, the Quarq and the FAD showed significant differences in the 500 W interval. Effect sizes were large when comparing the three power meters, except for the all-out sprints, which exhibited a medium effect size. When analyzing different power intervals across the power meters, effect sizes were large at 70 and 85 rev·min⁻^1^, medium at 100 rev min⁻^1^, and small when pedaling standing or during maximal sprints. Pearson and intraclass correlations between the FAD and the Quarq indicated a strong relationship (r > 0.883) and good reliability (ICC > 0.879), while the values, when comparing them to the H3, were considerably lower, except for the sprints, maximum power, and the 500 W interval (r > 0.843, ICC > 0.659) (Table 1).

**Figure 2:**
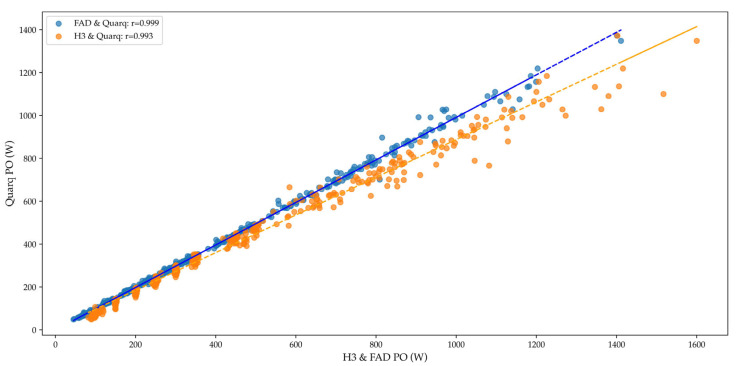
Correlation between power meters. Note: FAD, Favero Assioma Duo; H3, Hammer Saris H3; PO, power output, W, watts.

When analyzing all power data together, Pearson and intraclass correlations were extremely strong in both cases (r = 0.999 and 0.993; ICC = 0.999 and 0.986) (Figure 2). A repeated measures ANOVA revealed significant differences between the power measurements from the three power meters (*p* < 0.001) with an effect size of ηp^2^ = 0.364.

The coefficient of variation (CV%) ranged from 0.62% to 4.89% for the power values recorded by the Quarq and the FAD, with higher values observed at 100 W during the first block and in sprints. For the Quarq and the H3, very high CV% values were noted at 100 W and during sprints or at maximal power, while the values were below 2% for the remaining intervals (Table 2).

**Figure 3:**
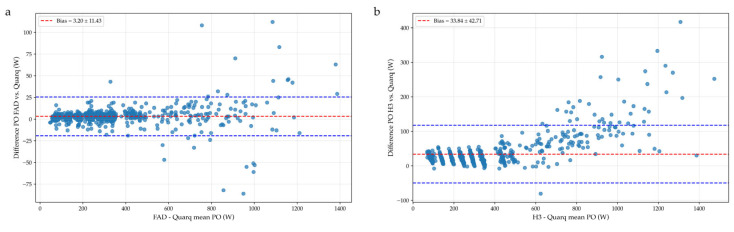
Bland–Altman diagram comparing power output measurements. (**a**) FAD vs. Quarq; (**b**) H3 vs. Quarq. Note: FAD, Favero Assioma Duo; H3, Saris H3; PO, power output; W, watts.

The Bland–Altman analysis showed no systematic bias between the FAD and the Quarq across intensities, and both devices exhibited similar values at all power levels (100 W to 1400 W), with a mean bias of 3.20 W (Figure 3a). As power levels increased, the limits of agreement (LoA) widened, indicating greater differences between devices, while the random bias also showed a slight increase. Specifically, a low bias between the power values of the FAD and the Quarq was observed during the first block (100 W to 500 W) at 70 rev·min⁻^1^, 1.7 W (LoA: −3.17 to 6.59), 1.9 W (LoA: −4.81 to 8.66) at 85 rev·min⁻^1^, and 3.2 W (LoA: −3.79 to 10.17) at 100 rev·min⁻^1^. Blocks 2 (bias: 4.2 W, LoA: −5.41 to 13.86), 3 (bias 5.37 W, LoA: −9.71 to 20.44), and 4 (3.3 W, LoA: −47.96 to 54.67) showed similar results. During the all-out 10-s sprint (fourth block), the bias was 11.9 W (LoA: 46.55–70.36), representing a 0.14% difference.

On the other hand, the Bland–Altman analysis revealed a higher bias between the Quarq and the H3 across all intensities (Figure 3b), indicating a less consistent agreement compared to the FAD and the Quarq measurements. In the first block, the bias was 13.6 W (LoA: −3.68 to 30.90) at 70 rev·min⁻^1^, 20.5 W (LoA: 4.23 to 36.37) at 85 rev·min⁻^1^, 27.1 W (LoA: 8.30 to 45.98) at 100 rev·min⁻^1^. In blocks 2, 3, and 4, the bias values were 39.1 W, (LoA: −2.82 to 81.10), 21.8 W (LoA: −2.52 to 45.94), and 76.7 W (LoA: −2.31 to 155.84), respectively. The bias for peak PO was particularly high, reaching 179 W (LoA: −13.55 to 371.48), highlighting substantial measurement discrepancies at maximal effort levels.

## 4. Discussion

The main objective of this study was to assess the reliability and validity of the Quarq power meter in measuring power output across different intensities and cadences. Results indicate that the Quarq power meter provides consistent and reliable measurements across various intensities and cadences. This study represents the first validation of the Quarq’s accuracy, noting challenges in comparing it to the SRM power meter, considered the gold standard, due to their shared mounting location on the bike (spider). In contrast, previous studies have typically validated power meters using devices located in different positions, such as rear hub-based systems [17] or ergometers [18], to avoid this potential source of measurement redundancy.

Post hoc analyses using the Bonferroni method confirmed that power measurements from the Quarq and the FAD were similar, with no significant differences except for certain high-intensity intervals. These findings support the reliability and accuracy of the Quarq’s measurements. Previous validation studies [9,10] yield consistent results between the FAD and the SRM, reinforcing the conclusions of this study. However, significant differences were observed between the power outputs of the H3 and the Quarq, with the former showing lower values. During the validation of this device [2], significant differences were found at certain power levels and cadences compared to the SRM.

The observed bias between power meters was minimal, with a reduced bias (<4 W) for power stages up to 350 W across different cadences. However, during all-out sprints, the bias FAD-Quarq increased slightly (<7 W), with the FAD measuring higher values. This small bias, particularly during maximum efforts, may be due to device placement differences and mechanical power transmission variances (e.g., crank deformation and pedal-crank interface losses). Similar results have been reported in previous studies [19] comparing the FAD and the SRM. Consistent with the findings of Fremeaux et al. [20], the H3 recorded higher power values compared to power meters positioned in the spider.

The study observed that the FAD tends to record higher power values due to its placement. Previous studies [21,22,23] comparing power meters (e.g., PowerTap, Stages, Garmin Vector) with the SRM found that pedal-mounted devices (Garmin Vector) and crank-mounted devices (Stages) typically reported higher values, while hub-mounted ones (PowerTap G3) recorded lower values. These findings align with the present study, where the FAD showed slightly higher power than the Quarq, with a similar coefficient of variation (CV) of 2.4% [22]. This discrepancy may be partly attributed to crank deformation, particularly in carbon cranks, which could explain the lower power recorded by the Quarq. However, another study [24] reported that the SRM measured higher power than the pedal-mounted PowerTap P1. Additionally, other research [10] noted that the SRM tends to yield higher readings than the FAD, potentially due to differences in strain gauge sensitivity or signal processing.

Regarding the CV for FAD–Quarq, values remained low (<4%) across most tests, with no cases exceeding 5%. A previous study [9] reported similar low CVs (<2.82%), except during sprints. Yeh et al. (2022) [19] also found mean biases of 3.6%, aligning with the present findings. According to Hopkins [25], CVs below 5% are generally acceptable in sports science. In this study the overall CV between the FAD and the Quarq was 3.6%, with a bias of 3.1 W ± 11.6 W, indicating strong precision and consistency. The importance of using reliable devices to detect small performance changes was been emphasized by Hopkins et al. [14]. Despite the stricter criterion for elite performance analysis (CV below 2%) [26] the Quarq still demonstrates valid and reliable data. These findings ensure that users can rely on the consistency of their daily training measurements and that any changes in power output reflect real performance variations. Furthermore, the precision values for 54 power meters (including pedal-based, crank arm, spider, and wheel hub models) reported in Maier et al.’s study [27] align with our results, showing CV values below 2%. Although the tests were conducted in a controlled indoor environment, the inclusion of both steady-state and high-intensity sprint efforts (up to 1400 W) ensured a broad representation of typical cycling demands [28,29], supporting ecological validity.

The ICC analysis showed excellent reliability (ICC > 0.90) for most tests, further supporting the Quarq’s validity in power measurement and analysis compared with the FAD. Similar to previous validation studies of power meters installed in pedals and spiders [9,10,22], this study also observed increasing typical errors proportional to average power, indicating greater precision at lower power levels compared to maximum power efforts (e.g., all-out and P_max_).

## 5. Conclusions

This study represents the first validation of the Quarq D-Zero power meter, representing a significant advance in this field. Comparing it with the gold standard (SRM) is challenging due to their shared placement on the bicycle (the crank spider). The analysis conducted in this study demonstrates that the Quarq power meter is a reliable and valid device for measuring cycling power, even under varying intensities, cadences, and cycling positions. The results indicate that the Quarq provides consistent and comparable power measurements to other devices, such as the FAD, particularly in intervals up to 350 W and maximum efforts. These findings establish the Quarq as a valuable tool for athletes and coaches aiming to monitor and optimize performance in training and competition, offering a robust and accurate alternative to existing reference measurement devices.

## Figures and Tables

**Table 1:** Analysis of the average and maximum power recorded by the Quarq Force, FAD, and H3 power meters at different cadences and intensities.

			QUARQ (W)	FAD (W)	H3 (W)	*p*-Value Power Meter	Effect Size ηp2 Power Meter	*p*-Value Bonferroni Power Meter		*p*-Value PO × Power Meter	Effect Size ηp2 PO × Power Meter	*p*-Value Bonferroni PO × Power Meter	
			Mean ± SD	Mean ± SD	Mean ± SD			Q-FAD	Q-H3			FAD-Q	H3-Q
**70 rev·min** ^−**1**^	**Sitting**	**100 W**	73.6 ± 12.7	74.7 ± 13.7	96.3 ± 4.6	<0.001	0.516	0.785	<0.001	<0.001	0.318	1.00	<0.001
		**150 W**	134.8 ± 7.2	136.9 ± 7.6	149.8 ± 0.8							1.00	<0.001
		**200 W**	184.3 ± 6.5	186.1 ± 6.8	199.9 ± 1.1							1.00	<0.001
		**250 W**	239.7 ± 6.7	241.3 ± 7.5	250.5 ± 1.1							1.00	<0.001
		**300 W**	290.3 ± 6.3	292.0 ± 7.2	299.9 ± 1.0							1.00	<0.001
		**350 W**	341.7 ± 6.8	343.7 ± 7.3	349.6 ± 1.6							1.00	<0.001
**85 rev·min** ^−**1**^	**Sitting**	**100 W**	74.2 ± 8.7	76.3 ± 8.5	99.0 ± 2.01	<0.001	0.732	0.539	<0.001	<0.001	0.115	1.00	<0.001
		**150 W**	129.5 ± 7.2	131.0 ± 7.4	150.4 ± 0.9							1.00	<0.001
		**200 W**	181.7 ± 6.7	183.4 ± 7.1	200.6 ± 1.4							1.00	<0.001
		**250 W**	228.2 ± 7.8	230.3 ± 8.1	250.4 ± 1.2							1.00	<0.001
		**300 W**	281.5 ± 8.4	283.7 ± 8.5	300.1 ± 1.5							1.00	<0.001
		**350 W**	333.4 ± 8.6	335.3 ± 9.1	349.7 ± 2.2							1.00	<0.001
**100 rev·min** ^−**1**^	**Sitting**	**100 W**	82.5 ± 6.5	85.3 ± 7.3	104.7 ± 6.5	<0.001	0.776	0.181	<0.001	<0.001	0.077	1.00	<0.001
		**150 W**	124.7 ± 11.8	127.5 ± 11.5	150.3 ± 1.2							1.00	<0.001
		**200 W**	172.4 ± 9.3	175.4 ± 9.0	200.5 ± 0.9							1.00	<0.001
		**250 W**	223.7 ± 8.9	227.4 ± 8.6	250.5 ± 1.6							1.00	<0.001
		**300 W**	269.4 ± 9.5	273.0 ± 9.5	300.5 ± 1.8							1.00	<0.001
		**350 W**	320.5 ± 10.3	323.8 ± 11.2	349.7 ± 2.2							1.00	<0.001
**Free**	**Sitting**	**500 W**	450.3 ± 32.6	454.5 ± 31.5	489.5 ± 15.3	<0.001	0.758	<0.001	<0.001	<0.001	-	-	-
**Free**	**Standing**	**250 W**	227.2 ± 13.1	232.9 ± 14.9	249 ± 4.2	<0.001	0.413	0.322	<0.001	<0.001	0.004	1.00	<0.001
		**350 W**	327.4 ± 12.7	331.9 ± 14.5	348.4 ± 3.4							1.00	<0.001
		**450 W**	422.3 ± 18.1	426.6 ± 18.8	444.8 ± 8.7							1.00	<0.001
**Free**	**Sitting**	**10 s all-out**	775.0 ± 183.2	781.8 ± 182.8	846.0 ± 201.6	0.150	0.041	1.00	0.256	0.803	0.009	1.00	1.00
		**10 s all-out**	758.1 ± 165.5	757.9 ± 164.9	834.1 ± 185.3							1.00	1.00
		**10 s all-out**	750.5 ± 156.6	753.8 ± 161	833.7 ± 180.0							1.00	1.00
**Free**	**Sitting**	**P_max_**	894.4 ± 209.1	906.3 ± 221.9	1073.4 ± 262.3	<0.001	0.767	0.102	<0.001	<0.001	-	-	-

Note: FAD, Favero Assioma Duo; H3, Saris H3; P_max_, Maximum power output; PO, Power output; Q, Quarq; rev·min^—1^, revolutions per minute; s, seconds; SD, Standard Deviation; W, watts.

**Table 2:** Validity statistics between power meters.

			TEM (W)		CV%		Pearson *r*-Value		CCI		Bland–Altman (Bias ± DE Bias)	
			FAD-Q	H3-Q	FAD-Q	H3-Q	FAD-Q	H3-Q	FAD-Q	H3-Q	FAD-Q	H3-Q
**70 rev·min^−1^**	**Sitting**	**100 W**	2.73	3.5	3.68%	4.12%	0.981	0.662	0.979	0.429	1.1 ± 2.8 LoA (−4.3 to 6.5)	22.7 ± 10.3 LoA (−2.5 to 43.0)
		**150 W**	2.50	0.79	1.84%	0.55%	0.948	0.127	0.949	0.028	2.1 ± 2.5 LoA (−2.7 to 6.9)	15.0 ± 7.1 LoA (1.0 to 29.0)
		**200 W**	2.40	1.14	1.30%	0.60%	0.941	0.197	0.941	0.069	1.8 ± 2.36 LoA (−2.9 to 6.4)	15.6 ± 6.4 LoA (3.1 to 28.1)
		**250 W**	2.52	1.09	1.05%	0.45%	0.96	0.291	0.942	0.097	1.55 ± 2.50 LoA (−3.4 to 6.5)	10.8 ± 6.4 LoA (−1.9 to 23.4)
		**300 W**	2.67	0.99	0.92%	0.34%	0.933	0.077	0.927	0.024	1.7 ± 2.7 LoA (−3.5 to 7)	9.6 ± 6.3 LoA (−2.7 to 21.9)
		**350 W**	2.25	1.64	0.66%	0.47%	0.955	−0.009	0.954	−0.004	2.0 ± 2.22 LoA (−2.4 to 6.4)	7.9 ± 7.0 LoA (−5.8 to 21.6)
**85 rev·min^−1^**	**Sitting**	**100 W**	3.68	1.89	4.89%	2.20%	0.907	0.383	0.910	0.226	2.1 ± 3.8 LoA (−5.3 to 9.4)	25.9 ± 5.9 LoA (14.41 to 37.39)
		**150 W**	2.92	0.87	2.24%	0.62%	0.924	0.267	0.927	0.066	1.6 ± 2.9 LoA (−4.1 to 7.3)	20.7 ± 7.1 LoA (6.9 to 34.6)
		**200 W**	3.10	1.42	1.70%	0.74%	0.906	0.098	0.907	0.040	1.7 ± 3.1 LoA (−4.3 to 7.7)	18.9 ± 6.7 LoA (5.9 to 32)
		**250 W**	3.27	1.24	1.43%	0.52%	0.921	0.158	0.923	0.049	2.1 ± 3.2 LoA (−4.3 to 8.4)	22.3 ± 7.7 LoA (7.1 to 37.4)
		**300 W**	4.00	1.47	1.42%	0.51%	0.891	0.114	0.894	0.039	2.2 ± 4.0 LoA (−5.7 to 10.1)	18.6 ± 8.3 LoA (2.3 to 34.9)
		**350 W**	3.80	2.2	1.14%	0.64%	0.915	0.128	0.916	0.062	1.9 ± 3.8 LoA (−5.4 to 9.3)	16.3 ± 8.6 LoA (0.6 to 33.1)
**100 rev·min^−1^**	**Sitting**	**100 W**	3.51	6.17	4.19%	6.59%	0.884	0.377	0.882	0.383	2.74 ± 3.5 LoA (−4 to 9.5)	22.1 ± 7.3 LoA (7.83 to 36.4)
		**150 W**	3.66	1.05	2.9%	0.76%	0.951	0.543	0.953	0.114	2.8 ± 3.7 LoA (−4.4 to 10.1)	25.6 ± 11.2 LoA (3.7 to 47.5)
		**200 W**	3.04	0.93	1.75%	0.50%	0.945	0.180	0.947	0.036	3.0 ± 3.1 LoA (−3.1 to 9.1)	28.1 ± 9.2 LoA (10.1 to 46.2)
		**250 W**	3.90	1.59	1.73%	0.67%	0.899	0.099	0.902	0.035	3.7 ± 4.0 LoA (−4.1 to 11.6)	26.8 ± 8.9 LoA (9.4 to 44.2)
		**300 W**	3.52	1.81	1.30%	0.64%	0.934	0.256	0.936	0.098	3.5 ± 3.5 LoA (−3.4 to 10.4)	31.0 ± 9.2 LoA (13 to 49.1)
		**350 W**	3.73	2.14	1.17%	0.64%	0.946	0.356	0.944	0.151	3.3 ± 3.7 LoA (−3.9 to 10.6)	29.2 ± 9.7 LoA (10.1 to 48.2)
**Free**	**Sitting**	**500 W**	4.80	8.5	1.06%	1.81%	0.989	0.843	0.989	0.659	4.2 ± 4.9 LoA (−5.4 to 13.9)	39.1 ± 21.4 LoA (−2.8 to 81.1)
**Free**	**Standing**	**250 W**	5.61	3.18	2.44%	1.33%	0.931	0.666	0.926	0.392	5.7 ± 5.6 LoA (−5.2 to 16.7)	21.8 ± 10.8 LoA (0.6 to 42.9)
		**350 W**	7.02	2.61	2.13%	0.77%	0.883	0.666	0.879	0.341	4.5 ± 6.9 LoA (−9.0 to 18.0)	21.00 ± 10.8 LoA (−0.1 to 42.1)
		**450 W**	6.86	7.32	1.62%	1.69%	0.935	0.576	0.937	0.458	4.3 ± 6.8 LoA (−9.0 to 17.6)	22.5 ± 15.0 LoA (−6.8 to 51.9)
**Free**	**Sitting**	**10-s all-out**	33.01	45.31	4.24%	5.59%	0.985	0.976	0.985	0.973	6.8 ± 32.6 LoA (−57.1 to 70.8)	71.0 ± 46.5 LoA (−20.2 to 162.2)
		**10-s all-out**	22.22	34.15	2.93%	4.29%	0.991	0.984	0.992	0.979	−0.2 ± 21.9 LoA (−43.2 to 42.8)	76.0 ± 37.5 LoA (2.5 to 149.6)
		**10-s all-out**	23.25	30.56	3.09%	3.86%	0.990	0.986	0.990	0.978	3.4 ± 23.0 LoA (−41.53 to 48.5)	83.2 ± 36.6 LoA (11.5 to 155.0)
**Free**	**Sitting**	**P_max_**	28.16	92.57	3.13%	9.41%	0.992	0.940	0.991	0.920	11.90 ± 29.8 LoA (−46.6 to 70.4)	179 ± 98.2 LoA (−13.5 to 371.5)

Note: FAD, Favero Assioma Duo; H3, Saris H3; P_max_, Maximum power output; rev·min^−1^, revolutions per minute; s, seconds; W, watts.

## Data Availability

Data are contained within the article.
